# Gene Expression Profiling in Ovaries and Association Analyses Reveal *HEP21* as a Candidate Gene for Sexual Maturity in Chickens

**DOI:** 10.3390/ani10020181

**Published:** 2020-01-21

**Authors:** Biao Chen, Guitao Liang, Xuenong Zhu, Yuwen Tan, Jiguo Xu, Hongxiang Wu, Huirong Mao, Yutao Zhang, Jiakun Chen, Yousheng Rao, Min Zhou, Sanfeng Liu

**Affiliations:** 1 College of Animal Science and Technology, Jiangxi Agricultural University, Nanchang 330045, Jiangxi, China; chenbiao@jxau.edu.cn (B.C.); lgt@besun.com.cn (G.L.); whxnc@aliyun.com (H.W.); huirongmjxau@126.com (H.M.); 2 Biotech Research Institute of Nanchang Normal University, Nanchang 330032, Jiangxi, China; zhuxuenong@163.com (X.Z.); yuwentan@126.com (Y.T.); xujiguo163@163.com (J.X.); zyt15297701409@163.com (Y.Z.); 13607066102@163.com (J.C.); rys8323571@aliyun.com (Y.R.); 3 Jiang Xi Province Key Lab of Genetic Improvement of Indigenous Chicken Breeds, Nanchang 330032, Jiangxi, China

**Keywords:** sexual maturity, chicken, *HEP21*, reproductive traits, digital gene expression RNA sequencing

## Abstract

**Simple Summary:**

Chicken meat and egg productions are essential for human beings. Sexual maturity is important for both egg production and meat flavor. It is necessary to elucidate the genetic mechanism of chicken sexual maturity. In current study, we used digital gene expression (DGE) RNA-sequencing analysis to investigate differential expression of genes in pre-pubertal and post-pubertal ovaries in two different sub-breeds of chicken with different onsets of sexual maturity. After the analysis of RNA-sequencing data, numerous differentially expressed genes were found in both comparisons (32 day old, early-sexual-maturity pre-laying hens (P-F-O1) vs. 103 day old early-sexual-maturity laying hens (P-F-O2), and 32 day old late-sexual-maturity pre-laying hens (L-F-O1) vs. 153 day old late-sexual-maturity pre-laying hens (L-F-O2)). With the bioinformatic analysis, hen egg protein 21 kDa (*HEP21*) was chosen as the candidate gene to conduct following experiment. The variations in *HEP21* were screened and association analyses between rs315156783 and reproductive traits were investigated in fifth-generation Ningdu Yellow chickens from a closely bred population. These results demonstrated that *HEP21* is a candidate gene for sexual maturity and ovary development in chickens. However, the underlying mechanism of how *HEP21* regulates chicken sexual maturity needs further focused studies.

**Abstract:**

The age of onset of sexual maturity is an important reproductive trait in chickens. In this study, we explored candidate genes associated with sexual maturity and ovary development in chickens. We performed DGE RNA-sequencing analyses of ovaries of pre-laying (P-F-O1, L-F-O1) and laying (P-F-O2, L-F-O2) hens of two sub-breeds of Ningdu Yellow chicken. A total of 3197 genes were identified in the two comparisons, and 966 and 1860 genes were detected exclusively in comparisons of P-F-O1 vs. P-F-O2 and L-F-O1 vs. L-F-O2, respectively. Gene ontology (GO) and Kyoto Encyclopedia of Genes and Genomes (KEGG) enrichment analyses showed that genes involved in transmembrane signaling receptor activity, cell adhesion, developmental processes, the neuroactive ligand–receptor interaction pathway, and the calcium signaling pathway were enriched in both comparisons. Genes on these pathways, including growth hormone (*GH*), integrin subunit beta 3 (*ITGB3*), thyroid stimulating hormone subunit beta (*TSHB*), prolactin (*PRL*), and transforming growth factor beta 3 (*TGFB3*), play indispensable roles in sexual maturity. As a gene unique to poultry, hen egg protein 21 kDa (*HEP21*) was chosen as the candidate gene. Differential expression and association analyses were performed. RNA-seq data and qPCR showed that *HEP21* was significantly differentially expressed in pre-pubertal and pubertal ovaries. A total of 23 variations were detected in *HEP21*. Association analyses of single nucleotide polymorphisms (SNPs) in *HEP21* and reproductive traits showed that rs315156783 was significantly related to comb height at 84 and 91 days. These results indicate that *HEP21* is a candidate gene for sexual maturity in chickens. Our results contribute to a more comprehensive understanding of sexual maturity and reproduction in chickens.

## 1. Introduction

In animals, sexual maturity is accompanied by aging, changes in tissue morphology, increased body weight, and reproductive competence [1]. Ovaries are the main reproductive organs of female animals. The structure of the ovaries changes dynamically during animal pubertal maturity, and these structural changes are controlled by nutrition, genetic factors, and concentrations of endocrine hormones [2]. Many studies have been performed to identify the key genes affecting sexual maturity. Multiple genes, quantitative trait loci (QTLs), and SNPs have been identified by RNA sequencing, genome-wide association studies, and genome-wide DNA methylation sequencing [3,4,5].

Chickens are a common domesticated animal with the largest breeding stock, and are gradually becoming the most important model in poultry research. The age of onset of sexual maturity is an important reproductive trait in chickens. Sexual maturity in chickens is associated with body weight [6,7]. In China, breeds with a younger age at first egg are more popular than those with an older age at first egg, because sexually mature chickens have relatively higher intramuscular fat deposition, which is correlated with flavor, juiciness, and tenderness of skeletal muscle [8,9]. However, the genetic mechanisms behind the onset of sexual maturity in chickens are still unclear, and more systematic research on the network of genetic factors responsible for this trait is needed. A previous study on ovaries in different stages of development showed that CCT6A might play a crucial role in sexual maturity in chickens [10].

In the present study, we used digital gene expression (DGE) RNA-sequencing analysis to investigate differential expression of genes in pre-pubertal and post-pubertal ovaries in two different sub-breeds of chickens with different onsets of sexual maturity. The Ningdu Yellow chicken is a Chinese breed characterized by early sexual maturity and meat with good flavor. This breed has been used for studies on reproductive traits in chickens, such as egg production and broodiness [11,12]. After RNA sequencing, we performed qPCR to validate the data, and gene ontology (GO) and Kyoto Encyclopedia of Genes and Genomes (KEGG) pathway analyses were also performed. The DGE RNA-seq data showed a large number of differentially expressed genes in the pre-pubertal and post-pubertal samples. We aim to investigate the differentially expressed genes and their functional pathways. The results may contribute to further understanding of the underlying mechanisms behind the reproductive process in chickens.

## 2. Materials and Methods

### 2.1. Ethics Statement

The animal experiments in this study complied with the ethical standards and regulations of Jiangxi Agricultural University (JXAULL-2017002). Effort was made to minimize the suffering of the chickens.

### 2.2. Animals and Tissues

Two sub-breeds of Ningdu Yellow chicken with different onsets of sexual maturity, provided by the College of Animal Science and Technology, Jiangxi Agricultural University, Nanchang, China, were used for the DGE RNA-seq in this study. Briefly, all chickens had free access to water and were fed a standard diet. Four groups of birds were used: 32 day old early-sexual-maturity pre-laying hens (P-F-O1), 103 day old early-sexual-maturity laying hens (P-F-O2), 32 day old late-sexual-maturity pre-laying hens (L-F-O1), and 153 day old late-sexual-maturity pre-laying hens (L-F-O2). All chickens were sacrificed and their ovaries were collected, quick-frozen in liquid nitrogen, and stored at −80 °C until RNA extraction.

To validate the differentially expressed gene from DGE sequencing, a dozen 143 day old Baier Yellow chickens—that is, six pre-laying hens and six laying hens—from the Baier Yellow Chicken Breeding Farm, Shangrao, China were also used. Hens were sacrificed painlessly and their ovaries were collected, frozen in liquid nitrogen, and kept at −80 °C.

Fifth-generation Ningdu Yellow chickens from a closely bred population from Guangdong Wens Foodstuff Company, Guangdong, China, were used to analyze associations between SNPs and reproductive traits. All birds were fed and immunized following the standard procedure of Guangdong Wens Foodstuff Company. After rearing, chickens were raised individually, and all egg production and reproductive traits were recorded, including age at first egg; body weight and comb height (measuring the distance from the root of comb to the peak of the highest sawtooth of the comb) at 77, 84, and 91 days; weight of first egg; and the total egg amount from the age at first egg to 300 days old. Samples of genomic DNA, which were used for genotyping and association analyses, were extracted from EDTA-anticoagulated blood samples of 1300 hens.

### 2.3. RNA Extraction and Sequencing

We extracted total RNA from the ovaries of chickens using Trizol reagent (TaKaRa, Otsu, Japan) according to the manufacturer’s instructions. We evaluated RNA degradation by loading on 1% agarose gels. The concentration and quality of all RNA samples were examined with a NanoDrop 2000 spectrophotometer (NanoDrop, Wilmington, DE, USA). All samples were kept at −80 °C.

Subsequently, five ovary RNA libraries of the same group were mixed in equal amounts. Four pooled samples (P-F-O1, P-F-O2, L-F-O1, and L-F-O2) were produced in this way and then sent to Shanghai Majorbio Bio-Pharm Biotechnology (Shanghai, China) for DGE RNA sequencing. Briefly, cDNA libraries were prepared based on Illumina’s protocols, and each library was sequenced with the Illumina Hiseq 2000 (Illumina, San Diego, CA, USA) to obtain paired-end 21 bp reads. The rest of the cDNA libraries were stored at −80 °C for qPCR validation of differential gene expression.

### 2.4. Bioinformatics Analysis of DGE RNA Sequencing

All sequencing data were submitted to the Gene Expression Omnibus with accession number GSE136329. For raw data from RNA sequencing, we used SeqPrep (https://github.com/jstjohn/SeqPrep) and Sickle (https://github.com/najoshi/sickle) to remove reads containing adapters, unknown bases, and low-quality bases to obtain high-quality reads. We then used TopHat (http://tophat.cbcb.umd.edu/) to align the clean reads with the chicken reference genome Gallus_gallus-4.0/galGal4 (http://asia.ensembl.org/Gallus_gallus/Info/Index). Based on the mapped reads, genes were annotated, and the expression of all genes was calculated using fragments per kilobase of transcript per million (FPKM) mapped reads. We analyzed the differential expression of P-F-O1 and P-F-O2 and of L-F-O1 and L-F-O2 using Cuffdiff (http://cufflinks.cbcb.umd.edu/); genes with greater than two-fold changes between the two samples (FPKM ≥ 0.3 and |log_2_FC| ≥ 1) or *p* < 0.05 were considered differentially expressed. We calculated the false discovery rate to judge the significance of the difference in gene expression. Using Goatools (https://github.com/tanghaibao/GOatools), we subjected all expressed genes to GO analysis with Bonferroni-adjusted *p* < 0.05. Using KOBAS (http://kobas.cbi.pku.edu.cn/home.do), we performed KEGG pathway enrichment analyses of all differentially expressed genes (pathways with *p* < 0.05 were considered significantly enriched).

### 2.5. Complementary DNA (cDNA) Synthesis and Quantitative Real-Time PCR (qRT-PCR)

We reverse-transcribed the total RNA extracted from ovaries using the Primescript RT reagent kit with gDNA eraser (perfect real time; TaKaRa, Otsu, Japan) with a random primer, per the manufacturer’s instruction. We then diluted synthesized cDNA in RNase-free water at a ratio of 1:4. Relative mRNA expression was detected by qRT-PCR using SsoFast EvaGreen Supermix (Bio-Rad, Hercules, CA, USA). The β-actin gene was used as an internal control. qRT-PCR was performed on a CFX96 system (Bio-Rad) in a total volume of 20 µL:10 µL SsoFast EvaGreen Supermix, 0.5 µL each primer (10 µM), 8.0 µL RNase-free water, and 1 µL cDNA. The PCR procedures were as follows: 39 cycles at 94 °C for 2 min, 94 °C for 15 s, and Tm °C for 30 s; fluorescence was then determined at 65 °C to 95 °C. Each sample was examined in triplicate, and the expression fold was calculated using the comparative 2^−∆∆Ct^ method. All primers used in this study were designed with Premier Primer 5.0 (Premier Biosoft International, Palo Alto, CA, USA) and synthesized with Sangon (Sangon Biotech, Shanghai, China). Details on all primers used in this study are shown in Supplementary Table S1.

### 2.6. Polymorphisms in HEP21 and rs315156783 Associations with Reproductive Traits

Based on the sequence of *HEP21* (NCBI accession number: NM_204521.2) in Genbank, primers P1 to P5 were used to amplify *HEP21*. Twenty samples of genomic DNA from fifth-generation Ningdu Yellow chicken were used to identify variations in *HEP21* by direct sequencing. We analyzed sequences using DNASTAR (http://www.dnastar.com). Variations that occurred more than twice were regarded as mutations. The SNP rs315156783 was recognized by the restriction enzyme *Kpn I.* Primer P6 was used for subsequent association analyses of fifth-generation Ningdu Yellow chickens by polymerase chain reaction/restriction fragment length polymorphism (PCR-RFLP). All PCR products were digested by *Kpn I* (Femantas, Carlsbad, CA, USA) at 37 °C over 2 h and then loaded on 1.5% agarose gels for genotyping. Association analyses of rs315156783 and reproductive traits were conducted with SAS 9.0 (SAS Institute, Cary, NC, USA) with the following general linear model.

Y = μ+G+D+H+e

where *Y* represents the trait’s phenotypic value, *μ* represents the overall population mean, *G* represents the fixed effect of genotype, *D* represents the random effect of dam, H represents the fixed effect of hatch, and *e* represents the random residual.

## 3. Results

### 3.1. Assembly and Mapping Analyses of RNA-seq Reads

**Figure 1:**
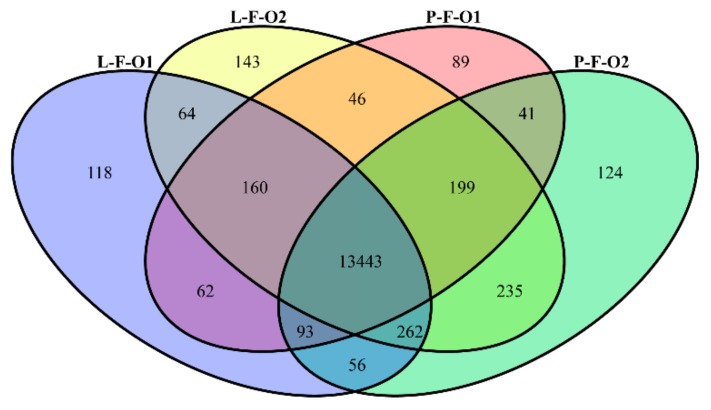
Numbers of expressed genes. Genes expressed in four groups. P-F-O1, P-F-O2, L-F-O1, and L-F-O2 denote 32 day old early-sexual-maturity pre-laying hens, 103 day old early-sexual-maturity laying hens, 32 day old late-sexual-maturity pre-laying hens, and 153 day old late-sexual-maturity pre-laying hens, respectively.

In this study, four libraries, P-F-O1, P-F-O2, L-F-O1, and L-F-O2, were constructed for RNA sequencing. After sequencing, more than 8 million reads were obtained from each sample. As shown in Table 1, 8,967,278, 16,977,869, 10,388,655, and 18,324,325 reads were obtained from P-F-O1, P-F-O2, L-F-O1, and L-F-O2, respectively. After the removal of low-quality reads and adapters, between 8,332,975 and 14,950,291 clean reads were obtained from the four libraries (Table 1). Furthermore, the clean reads were aligned with the chicken reference genome. A total of 7,416,348 (89%), 11,056,434 (80%), 8,601,177 (89%), and 12,259,239 (82%) reads were mapped to the chicken genome. In total, 15,134 genes were detected in the four libraries, and 14,332, 14,653, 14,456, and 14,751 were identified in the libraries of P-F-O1, P-F-O2, L-F-O1, and L-F-O2, respectively (Figure 1). Of these genes, 13,443 were found in all four libraries, whereas 118, 143, 89, and 124 genes were detected only in P-F-O1, P-F-O2, L-F-O1, and L-F-O2, respectively (Figure 1).

### 3.2. Differential Gene Expression in Immature and Mature Chicken Ovaries

**Figure 2:**
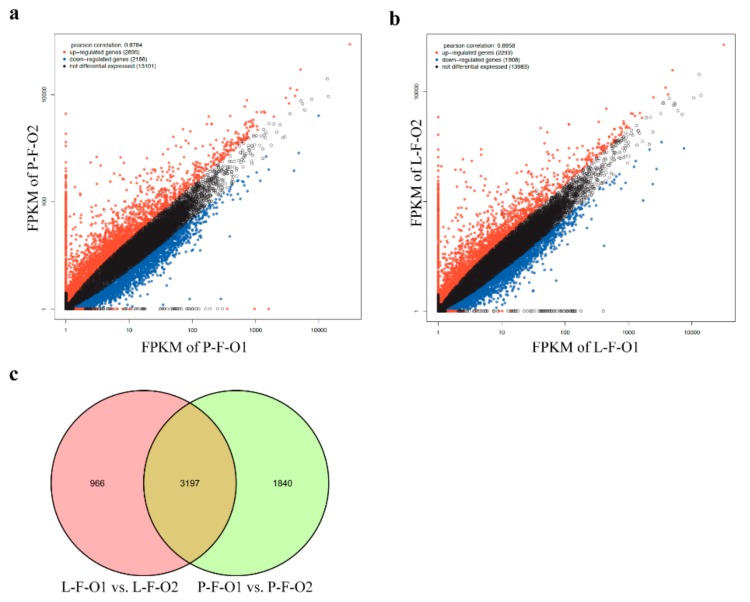
Two comparisons of differentially expressed genes. (**a**,**b**) Gene expression of P-F-O1/P-F-O2 and L-F-O1/L-F-O2. (**c**) Differentially expressed genes in comparisons of P-F-O1 vs. P-F-O2 and L-F-O1 vs. L-F-O2. P-F-O1, 32 day old early-sexual-maturity pre-laying hens; P-F-O2, 103 day old early-sexual-maturity laying hens; L-F-O1, 32 day old late-sexual-maturity pre-laying hens; L-F-O2, 153 day old late-sexual-maturity pre-laying hens.

To analyze differential gene expression in P-F-O1 and P-F-O2 (P-F-O1 vs. P-F-O2) and in L-F-O1 and L-F-O2 (L-F-O1 vs. L-F-O2), we compared the expression profiles of each group using a Poisson distribution model. The results showed that 5083 genes were differentially expressed in P-F-O1 vs. P-F-O2: 2895 were upregulated and 2188 were downregulated (|logFC| ≥ 1; Figure 2a). The full list of differentially expressed genes can be found in Supplementary Table S2. In the L-F-O1 vs. L-F-O2 comparison, 4201 genes were differentially expressed—2293 were upregulated and 1908 were downregulated (Figure 2b). Directionality analyses showed that there were more upregulated genes in both comparisons than downregulated genes. Of the differentially expressed genes, 3197 genes were found in both comparisons, whereas 966 and 1860 genes were detected exclusively in the L-F-O1 vs. L-F-O2 and P-F-O1 vs. P-F-O2 comparisons, respectively (Figure 2c).

### 3.3. Validation of Differential Gene Expression in RNA Sequencing

**Figure 3:**
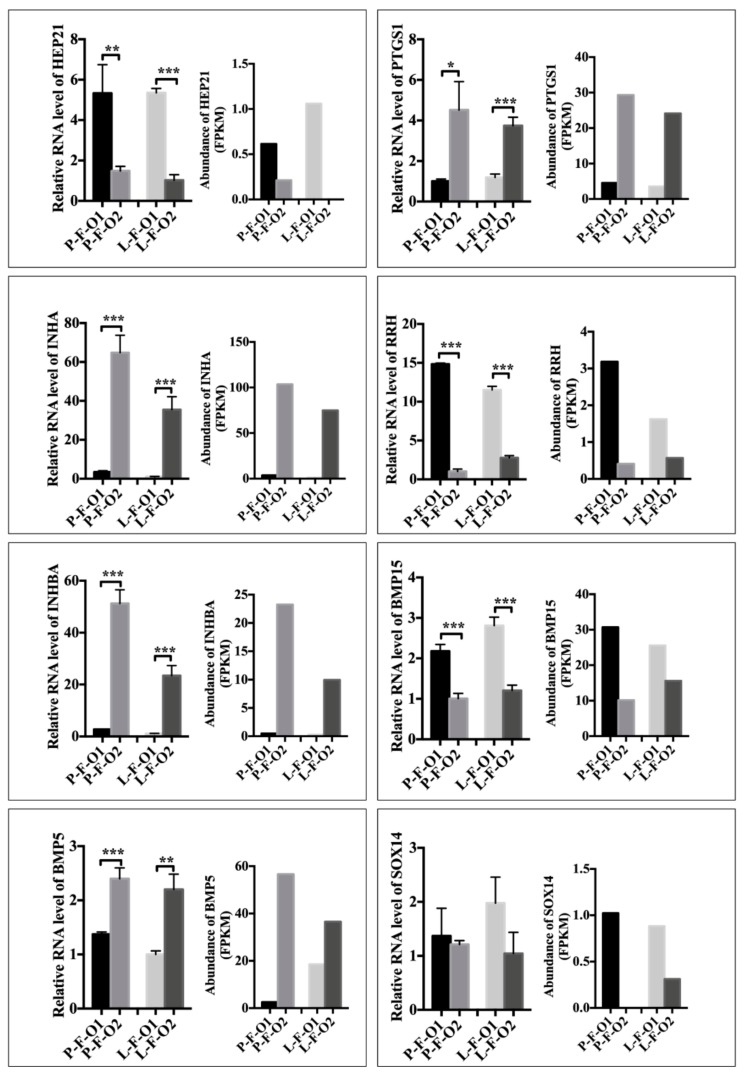
qRT-PCR validation of differentially expressed genes in RNA-seq. Each sample was examined in triplicate in qRT-PCR. Values represent means ± SEM based on qRT-PCR data. * *p* < 0.05; ** *p* < 0.01; *** *p* < 0.001. In RNA-seq data panels, values denote fragments per kilobase of transcript per million mapped reads of each gene.

To validate the differential gene expression in both comparisons of RNA sequencing, we randomly selected five genes with which to carry out qPCR using the same RNA samples used for sequencing. The five genes were *HEP21*, retinal pigment epithelium-derived rhodopsin homolog (*RRH*), inhibin subunit alpha (*INHA*), inhibin subunit beta A (*INHBA*), and prostaglandin-endoperoxide synthase 1 (*PTGS1*). The expression of the selected genes was in concordance with the results of sequencing in terms of the fold changes and the directions in each comparison (Figure 3). Two differentially expressed genes—bone morphogenetic protein 5 (*BMP5*) and bone morphogenetic protein 15 (*BMP15*)—from early sexual maturity and SRY-Box 14 (*SOX14*) from late sexual maturity were also chosen for qPCR to validate the results of sequencing, as these genes play crucial roles in ovary development and sex determination, respectively [13,14,15]. Results of qPCR showed that expression of *BMP5* and *BMP15* was in agreement with the results of RNA sequencing (Figure 3). Moreover, *BMP5* and *BMP15* were differentially expressed in both comparisons in our qPCR. However, the expression profile of *SOX14* differed from the results of RNA sequencing in its low endogenous expression (Figure 3).

**Figure 4:**
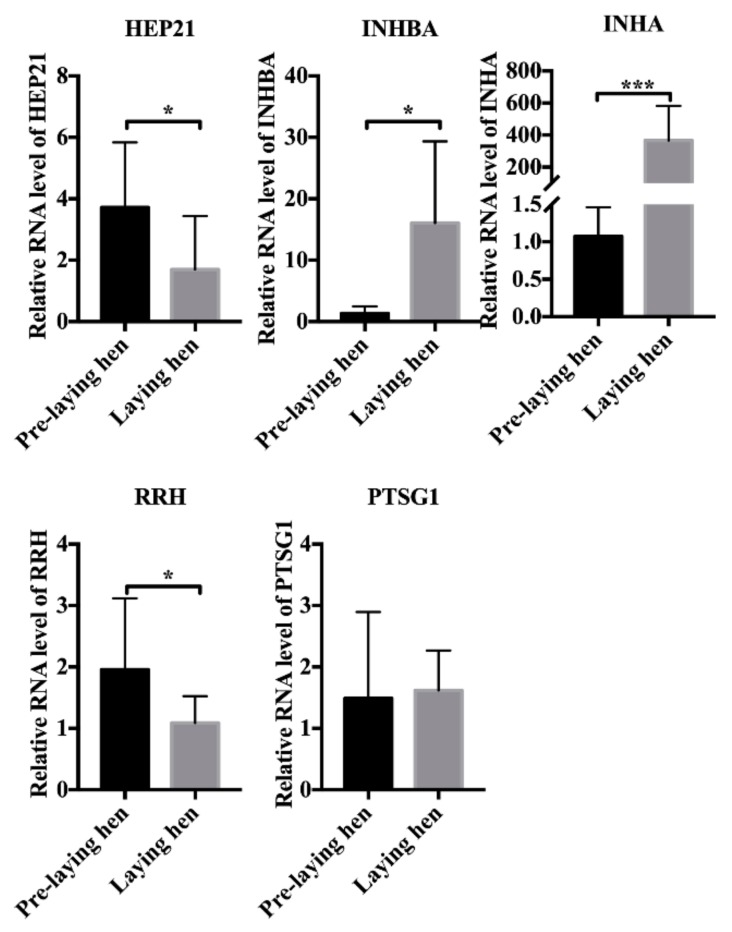
Differential gene expression in Baier Yellow chickens. cDNA from six chickens in each group was used as a template for qRT-PCR. Each sample was examined in triplicate. Values represent means ± SEM based on qRT-PCR data. * *p* < 0.05; *** *p* < 0.001.

To further investigate the role of differential gene expression in reproduction in chickens, we detected the expression of genes in ovaries of pre-laying and laying Baier Yellow hens. Results of qPCR showed that *HEP21*, *INHA*, *INHBA*, and *RRH* were markedly differentially expressed in the ovaries of pre-laying and laying hens, whereas *PGST1* was not differentially expressed (Figure 4). These results imply that *HEP21*, *INHA*, *INHBA*, and *RRH* might be indispensable to ovary development.

### 3.4. GO and KEGG Pathway Enrichment Analyses of Differential Gene Expression

**Figure 5:**
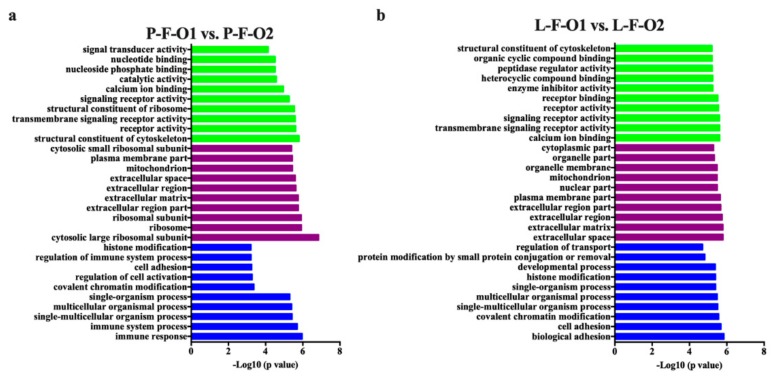
Gene ontology (GO) enrichment analyses of differentially expressed genes. (**a**,**b**) Top 10 enriched biological processes, cellular compounds, and molecular functions in P-F-O1 vs. P-F-O2 and L-F-O1 vs. L-F-O2. Biological process terms are in blue, cellular compound terms are in purple, and molecular function terms are in green. The Y axes represent GO terms, and the X axes represent −log *p* value.

To annotate the function of differentially expressed genes, we performed GO enrichment analyses (Bonferroni-adjusted *p* < 0.05). A total of 151 GO terms were identified, including 101 in P-F-O1 vs. P-F-O2, 123 in L-F-O1 vs. L-F-O2, and 73 in both comparisons (Supplementary Table S3). The top 10 significantly enriched biological processes, cellular compounds, and molecular functions are shown in Figure 5. Differentially expressed genes were mainly enriched in cell adhesion, developmental processes, receptor activity, calcium ion binding, transmembrane signaling receptor activity, and structural constituents of the cytoskeleton, which are linked to cell progression, metabolism, immunity, signaling pathways, and signaling response.

**Figure 6:**
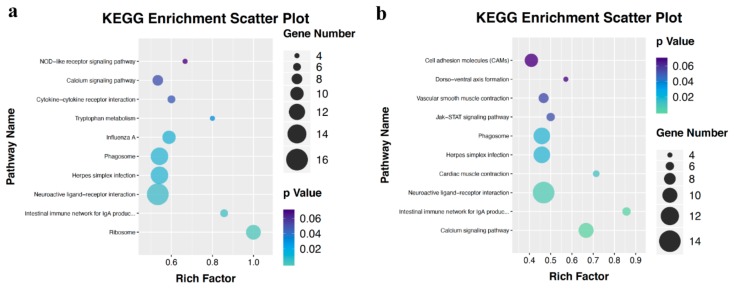
Kyoto Encyclopedia of Genes and Genomes (KEGG) pathway enrichment analyses of differentially expressed genes. (**a**,**b**) Top 10 enriched KEGG pathway in P-F-O1 vs. P-F-O2 and L-F-O1 vs. L-F-O2. The Y axes represent the name of pathway, the X axes represent richness factor (differentially expressed genes enriched in this pathway/total genes in this pathway).

To further investigate the biochemical pathways with the differentially expressed genes, we performed KEGG pathway analyses. There were nine and six pathways enriched with *p* < 0.05 in P-F-O1 vs. P-F-O2 and 123 in L-F-O1 vs. L-F-O2, respectively (Supplementary Table S4). Further analyses revealed five pathways in both comparisons: the intestinal immune network for IgA production, neuroactive ligand–receptor interaction, herpes simplex infection, phagosome, and calcium signaling pathways (Figure 6). Moreover, a total of 28 differentially expressed genes, including genes related to ovary development, such as *GH* [16], *ITGB3* [17], and *TSHB* [18], were detected in the five pathways (Table 2). Some genes that may be important to ovary development, that is, *PRL* [19] and *TGFB3* [20,21], were found in one of the two comparisons. These results indicate that the differentially expressed genes are mainly linked to cell metabolism, oocyte development, and signaling pathways.

### 3.5. Associations Between SNPs in HEP21 and Reproductive Traits in Chickens

The results from Figure 3 and Figure 4 and from a previous study [22] indicated that *HEP21* might play a crucial role in ovary development in chickens; as just a few studies had been published about *HEP21*, we selected it as our candidate gene. To screen for polymorphisms on *HEP21*, we designed five pairs of primers to amplify the gene sequence. After amplification and alignment of sequences, a total 23 mutations, that is, 22 SNPs and 1 indel, were identified in 2703 bp in the Ningdu Yellow chicken (Table 3). Of these variations, 11, 10, and 2 were located in the introns, flank region, and untranslated region (UTR) region of *HEP21*, respectively. It is interesting that no SNPs were found in the coding region, which indicates that *HEP21* is a relatively conserved gene.

We next found that the allele G of rs315156783 was recognized by restriction enzyme Kpn I but the allele A was not. Subsequently, genotypes AA and AG for rs315156783 were detected by PCR-RFLP with samples of genomic DNA from Ningdu Yellow chickens as a PCR template. The total amount of each genotype was counted; no genotype GG was identified. The frequency of genotypes AA and AG for rs315156783 is 0.283 and 0.717, respectively. Association analyses showed that rs315156783 was strongly associated with comb height at 84 days and comb height at 91 days (*p* < 0.05) but was not significantly associated with body weight at 77 days, body weight at 84 days, body weight at 91 days, comb height at 77 days, amount of eggs at 300 days, age at first egg, or weight of first egg (*p* > 0.05; Table 4). The mass of the comb is an important reproductive trait [23,24]. These results indicate that *HEP21* is a candidate gene related to chicken sexual maturity.

## 4. Discussion

The ovaries are key organs in the reproductive systems of chickens and play a decisive role in egg production and age at first egg. They can also secrete multiple kinds of hormones that exert important effects on the animal’s growth and development. In this study, to identify genes important to the sexual maturity of chicken, we investigated differences in transcriptome profiles in the ovaries of pre-laying and laying hens from two sub-breeds with different sexual maturity age using DGE RNA sequencing and identified genes involved in sexual maturity. We then detected genes differentially expressed in ovaries of pre-laying and laying hens of the same age. Finally, associations between SNPs in *HEP21* and reproductive traits were examined in a selected population of chickens.

After a long period of artificial selection, two sub-breeds of Ningdu Yellow chicken emerged. These two sub-breeds, with different body size, age of sexual maturity, and age of first egg provided an ideal model with which to research the chicken sexual maturity. In this study, these two sub-breeds of Ningdu Yellow chicken were used to investigate the crucial genes for sexual maturity.

A large number of differentially expressed genes were identified, and several GO terms and pathways were enriched in the present study. GO analyses showed that the differentially expressed genes were enriched in single-multicellular organisms, multicellular organisms, single organisms, and developmental processes associated with cell metabolism and tissue development. Some key genes in these groups may play crucial roles in ovary development. Like ETS transcription factor 3 (*ELF3*), E74 is a prognostic marker of ovarian cancer and can suppress the proliferation of ovarian cancer cells [25]. Adenosine A1 receptor (*ADORA1*) plays a role in fertilization by blocking adenylyl cyclase, and genome-wide transcriptome analysis has shown that *ADORA1* is a candidate gene for sexual precocity in goats [26]. Signaling receptor activity, in which an extracellular signal is received and transmitted across a membrane by activating an associated protein, was another enriched molecular function. A total of 619 differentially expressed genes were enriched in signaling receptor activity. Of these genes, corticotropin releasing hormone receptor 1 (*CRHR1*), platelet activating factor receptor (*PTAFR*), and 5-hydroxytryptamine receptor 4 (*HTR4*) are all highly expressed in ovaries, are involved in proliferation and apoptosis of ovarian cells, and play important roles in the development of the ovaries [27,28,29,30].

Results of the KEGG enrichment analyses showed that five pathways were significantly enriched in both comparisons: the intestinal immune network for IgA production, neuroactive ligand–receptor interaction, herpes simplex infection, phagosome, and calcium signaling pathways. The neuroactive ligand–receptor interaction and calcium signaling pathways are related to signaling molecules and interaction and signal transduction, respectively. GH and TSHB, which are important genes for growth and reproduction in poultry [31,32,33,34,35], are members of the neuroactive ligand–receptor interaction pathway. The intestinal immune network for the IgA production pathway is attached to the immune system. It is interesting that many interleukin members were differentially expressed in both comparisons. Both the hypothalamic–pituitary–gonad axis and immune system are complex and essential for the lives of animals, and more research is needed on system resources are balanced between reproduction and immunity [36].

Of the differentially expressed genes, bone morphogenetic protein 2 (*BMP2*), bone morphogenetic protein 3 (*BMP3*), and BMP binding endothelial regulator (*BMPER*) were identified in both comparisons; *BMP5* and *BMP15* were detected in P-F-O1 vs. P-F-O2 only (Supplementary Table S2). The bone morphogenetic protein system plays a crucial role in ovary development and the regulation of ovarian function [37]. Bone morphogenetic protein 6 (*BMP6*), a regulator associated with the formation and secretion of steroid hormones, can interact with melatonin [38,39]. Moreover, BMP2 and BMP15 are involved in the growth and differentiation of oocytes [40,41]. In this study, *BMP5* and *BMP15* were differentially expressed in P-F-O1 vs. P-F-O2 in the RNA-seq data but were significantly differentially expressed in both comparisons in qPCR validation. The results suggest that *BMP5* and *BMP15* might have strong effects on puberty and reproduction in chickens. INHA and INHBA have been implicated in regulating cell proliferation and hormone secretion and have been identified as candidate genes for abnormal ovarian development in female humans [42,43,44]. The proteins encoded by *INHA* and *INHBA* have indispensable functional roles in the recruitment and ordered progression of follicles in avian ovary development [45,46]. Our RNA-seq data showed that these two genes were significantly differentially expressed in the ovaries of pre-laying and laying hens, which implies that they might affect the precocity of chickens.

HEP21, a member of the uPAR/Ly6 protein superfamily, was first identified in 2003 and secreted into egg white [47]. HEP21 is mainly expressed in chicken oviducts and is affected by molting behavior [22]. The function of HEP21 is poorly understood. RNA-seq data and the results of qPCR in this study showed that *HEP21* was significantly differentially expressed in pre-pubertal and pubertal ovaries, which suggests that *HEP21* might play a crucial role in ovary development and the onset of puberty in chickens. Association analyses of SNPs in *HEP21* and reproductive traits showed that rs315156783 was significantly related to comb height at 84 and 91 days. Comb mass, which is an important reproductive trait, is a secondary sexual characteristic of chicken and associated with sexual maturity and reproduction in chickens [24]. rs315156783 may link to sexual maturity and reproduction in chickens through its association with comb height.

## 5. Conclusions

Collectively, these results provide a comprehensive transcriptome analysis of the ovaries of pre-laying and laying hens. *HEP21*, *INHA*, *INHBA*, *RRH*, *BMP5*, *BMP15, GH*, *ITGB3*, *TSHB*, and *TGFB3* may play important roles in sexual maturity in chickens. Association analyses demonstrated that *HEP21* is a candidate gene for sexual maturity. Our results contribute to a more comprehensive understanding of sexual maturity and reproduction in chickens.

## Figures and Tables

**Table 1:** Data generated from digital gene expression (DGE) RNA sequencing.

Sample	Total Number of Raw Reads	Total Number of Clean Reads	Total Residues (bp)	Total Mapped Reads	Percentage of Mapped Reads
P-F-O1	8,967,278	8,332,975	413,314,923	7,416,348	89%
P-F-O2	16,977,869	13,820,543	669,009,159	11,056,434	80%
L-F-O1	10,388,655	9,664,244	479,417,236	8,601,177	89%
L-F-O2	18,324,325	14,950,291	724,670,213	12,259,239	82%

Note: P-F-O1 represents ovary of early-sexual-maturity pre-laying hens; P-F-O2 represents ovary of early-sexual-maturity laying hens; L-F-O1 represents ovary of late-sexual-maturity pre-laying hens; L-F-O2 represents ovary of late-sexual-maturity laying hens.

**Table 2:** Differentially expressed genes enriched in the five pathways from P-F-O1 vs. P-F-O2 and L-F-O1 vs. L-F-O2.

Gene	Description	Gene	Description
*MHCDMA*	major histocompatibility complex, class II, DM alpha	*ADRA1B*	adrenoceptor alpha 1B
*MAP3K14*	mitogen-activated protein kinase kinase kinase 14	*AVPR1B*	arginine vasopressin receptor 1B
*BLB1*	Major histocompatibility complex class II beta chain BLB1	*TSHB*	thyroid stimulating hormone beta
*PIGR*	polymeric immunoglobulin receptor	*GABRA1*	gamma-aminobutyric acid type A receptor alpha1 subunit
*IL10*	interleukin 10	*PTAFR*	platelet activating factor receptor
*HTR4*	5-hydroxytryptamine receptor 4	*C5*	complement component 5
*ADORA1*	adenosine A1 receptor	*SRSF6*	serine and arginine rich splicing factor 6
*P2RX1*	purinergic receptor P2X 1	*ITGB3*	integrin subunit beta 3
*GH*	growth hormone	*ATP6V1G3*	ATPase H+ transporting V1 subunit G3
*CRHR1*	corticotropin releasing hormone receptor 1	*NCF1*	neutrophil cytosolic factor 1C pseudogene
*GRM4*	glutamate receptor, metabotropic 4	*CTSS*	cathepsin S
*GALR2*	galanin receptor 2	*SFTPA2*	surfactant protein A2
*ADORA3*	adenosine A3 receptor	*ATP2A3*	ATPase sarcoplasmic/endoplasmic reticulum Ca2+ transporting 3
*GABRB2*	gamma-aminobutyric acid type A receptor beta2 subunit	*TNNC1*	troponin C1, slow skeletal and cardiac type

**Table 3:** Mutations detected in *HEP21* gene from Ningdu Yellow chicken.

dbSNP rs id	Allele	Position on Chromosome 16	Variation Type	Region
rs315385113	CT	2494834	SNP	5’flank region
rs15787981	A→G	2494758	SNP	5’flank region
rs15787979	A→T	2494734	SNP	5’flank region
rs316912785	A→G	2494703	SNP	5’flank region
rs15787975	A→G	2494430	SNP	5’flank region
rs15787973	A→G	2494412	SNP	5’flank region
rs740811206	A→G	2494379	SNP	5’flank region
rs741766790	C→T	2494134	SNP	5’UTR
rs315616343	A→T	2494021	SNP	Intron1
rs317564949	C→T	2493983	SNP	Intron1
rs731677543	A→G	2493976	SNP	Intron1
rs314122647	C→T	2493760	SNP	Intron1
rs733275244	A→G	2493537	SNP	Intron2
rs317970605	C→T	2493510	SNP	Intron2
rs314173365	A→G	2493499	SNP	Intron2
rs313113635	C→G	2493260	SNP	Intron3
rs315156783	A→G	2493169	SNP	Intron3
rs316109464	A→G	2493165	SNP	Intron3
rs1058822254	A/-	2493123	Indel	Intron3
rs15026706	A→C	2492682	SNP	3’ UTR
rs1058676726	C→G	2492656	SNP	3’flank region
rs314965744	C→T	2492411	SNP	3’flank region
rs317030330	C→G	2492131	SNP	3’flank region

**Table 4:** Associations between rs315156783 and chicken reproductive traits.

Traits	*p*-Value	Genotype/Number	
		AA (n = 360)	AG (n = 912)
BW77	0.6489	834.22 ± 3.21	835.94 ± 2.02
CH77	0.1556	20.82 ± 0.28	21.28 ± 0.17
BW84	0.3181	892.12 ± 4.03	896.89 ± 255
CH84	0.0365	24.10 ± 0.29 *	24.82 ± 0.19 *
BW91	0.5520	935.69 ± 4.43	938.79 ± 2.77
CH91	0.0299	26.20 ± 0.30 *	26.97 ± 0.19 *
EA300	0.1201	95.52 ± 1.30	93.13 ± 0.82
AFE	0.0822	122.46 ± 0.850	124.21 ± 0.53
WFE	0.1034	27.65 ± 0.25	28.14 ± 0.16

Note 2: BW77, BW84, and BW91 represent body weight at 77, 84, and 91 days; CH77, CH84, and CH91 represent comb height at 77, 84, and 91 days; EA300 represents the egg amount of the hen at 300 days; AFE represents the age at first egg; WFE represents the weight of first egg. * *p* < 0.05.

## Supplementary Materials

The following are available online at https://www.mdpi.com/2076-2615/10/2/181/s1, Table S1: Primer information, Table S2: List of all genes, Table S3: List of all GO terms enriched, Table S4: Significantly enriched KEGG pathways.
