# Supplementary material for: Gene Expression Profiling in Ovaries and Association Analyses Reveal HEP21 as a Candidate Gene for Sexual Maturity in Chickens
Source: Animals (Basel). 2020 Jan 21;10(2):181. doi: 10.3390/ani10020181 (PMC7071030; doi:10.3390/ani10020181)
Supplement: Supplementary file 1 [file animals-10-00181-s001.zip › Supplementary Table 1.docx]

| Primer name | Sequence (5’-3’) | Tm (°C) | Product size (bp) | Application |
| --- | --- | --- | --- | --- |
| *BMP5* | F:GGATTATTGCACCAGAGGGCTATG | 60 | 181 | qPCR for BMP5 |
|  | R:AGCACGGAGATGGCATTCAG |  |  |  |
| *BMP15* | F:TCGCAGTCTGAGCACCAACAC | 60 | 150 | qPCR for BMP15 |
|  | R:TAGGCCACGGTGACACGGAGAAG |  |  |  |
| *INHA* | F:CCCAAAAGGATGTGAGGAGG | 60 | 185 | qPCR for INHA |
|  | R:CGAGGGCTGGAAGAGGTAAGTG |  |  |  |
| *INHBA* | F:GTGGGAGATGATGGCTATGTGG | 60 | 262 | qPCR for INHBA |
|  | R:GCTGCCGCTGCTGTTGAAAC |  |  |  |
| *PTGS1* | F:GGCTGTTGGAGTCATTGAGGAGTC | 60 | 207 | qPCR for PTGS1 |
|  | R:CCGTTGGGTTGGGGTTTCTC |  |  |  |
| *RRH* | F:CCTTGCATGGAAGCTGGAAGTTTGG | 60 | 173 | qPCR for RRH |
|  | R:GAGCGGCATAGTTACGGGTAGTC |  |  |  |
| *HEP21* | F:TGTGCGTTGGAGTGGTGAAGC | 60 | 225 | qPCR for HEP21 |
|  | R:CGGGATGTCAAACGGGATTC |  |  |  |
| *SOX14* | F:CGCAAGATGGCCCAGGAGAAC | 60 | 230 | qPCR for SOX14 |
|  | R:CCAGGTAAGGCAAAGGGAAGAC |  |  |  |
| *β-actin* | F: CTCCCCCATGCCATCCTCCGTCTG | 52-65 | 179 | qPCR for β-actin |
|  | R: GCTGTGGCCATCTCCTGCTC |  |  |  |
| P1 | F:AGCCCAGAACCCGATGATGAAG | 62 | 1049 | Amplification of partial sequence of *HEP21* |
|  | R:GGCAAACACAGCACGGAGAG |  |  |  |
| P2 | F:CAGCCCAAGAACAGCCTCATAAC | 56 | 925 |  |
|  | R:AGCCCTCGTTAACTCACCCACTC |  |  |  |
| P3 | F:CTGTGCGTTGGAGTGGGTGAG | 56 | 718 |  |
|  | R:CGGGATGTCAAACGGGATTC |  |  |  |
| P4 | F:TCCCGGTATTTCCTGCTGCGAGAC | 62 | 984 |  |
|  | R:CCCCAATCACACCTTTACTGCAG |  |  |  |
| P5 | F:ACGGGAGCTTGGATTTGCATTGG | 58 | 439 |  |
|  | R:ACCGCTCTCCGCTCTGCTCT |  |  |  |
| P6 | F:TGCGAGCGGAGGGAGAGATGT | 58 | 373 | Amplification of partial sequence of HEP21 which containing A+550G |
|  | R:CGGATGTCAAACGGGATTC |  |  |  |
